# Evidence for a mixed-age group in a pterosaur footprint assemblage from the early Upper Cretaceous of Korea

**DOI:** 10.1038/s41598-022-14966-5

**Published:** 2022-06-23

**Authors:** Jongyun Jung, Min Huh, David M. Unwin, Robert S. H. Smyth, Koo-Geun Hwang, Hyun-Joo Kim, Byung-Do Choi, Lida Xing

**Affiliations:** 1 Department of Geological and Environmental Sciences and Korea Dinosaur Research Center and Mudeungsan Geotourism Center, Chonnam National University, Gwangju, 61186 Republic of Korea; 2 School of Museum Studies, University of Leicester, 19 University Road, Leicester, LE1 7RF UK; 3 School of Geography, Geology and the Environment, University of Leicester, University Road, Leicester, LE1 7RH UK; 4 Yeosu Samil Middle School, Yeosu, 59655 Republic of Korea; 5 Division of Earth and Environmental System Sciences, Pukyong National University, Busan, 48513 Republic of Korea; 6 Daegu National Science Museum, Daegu, 43023 Republic of Korea; 7 School of the Earth Sciences and Resources, China University of Geosciences, Beijing, 100083 China

**Keywords:** Palaeontology, Behavioural ecology

## Abstract

Here we describe a new pterosaur footprint assemblage from the Hwasun Seoyuri tracksite in the Upper Cretaceous Jangdong Formation of the Neungju Basin in Korea. The assemblage consists of many randomly oriented prints in remarkably high densities but represents a single ichnotaxon, *Pteraichnus*. Individuals exhibit a large but continuous size range, some of which, with a wingspan estimated at 0.5 m, are among the smallest pterosaurs yet reported from the Upper Cretaceous, adding to other recent finds which contradict the idea that large and giant forms entirely dominated this interval. Unusual features of the tracks, including relatively long, slender pedal digit impressions, do not match the pes of any known Cretaceous pterosaur, suggesting that the trackmakers are as yet unknown from the body fossil record. The Hwasun pterosaur footprints appear to record gregarious behavior at the exact location by individuals of different ages, hinting at the possibility that pterosaurs gathered in mixed-age groups.

## Introduction

Pterosaurs, extinct flying archosaurs with a fossil record that extends from the Late Triassic to the terminal Cretaceous, were the first powered-flying vertebrates, appearing much earlier than birds, the only other archosaurs capable of powered-flight^1–3^. The most undersized known individuals, likely to be hatchlings but flight capable, had wingspans of only 0.175 m, while the largest had a wingspan of 10 m^4^. These flying reptiles were a principal component of the Mesozoic ecosystems^5^.

Pteraichnites, the tracks and traces produced by pterosaurs^2^, provide an alternative and essential source of data on behavior and ecology and enjoy several advantages over body fossils, for example, capturing movement, behaviors, and associations at a single point in time and a single location. So far, however, the vast majority of tracksites have been recovered from Upper Jurassic and Lower Cretaceous sequences.

Here, we report on a new assemblage of pterosaur footprints from the Hwasun Seoyuri tracksite, which forms part of the Upper Cretaceous Jangdong Formation of the Neungju Basin, Korea. This site has yielded numerous footprints made by individuals ranging from 0.5 to 1.5 m in wingspan, representing a not yet known from the body fossil record of pterosaurs. This find provides new evidence supporting the idea that Late Cretaceous pterosaur communities were not entirely composed of large and giant individuals but also included smaller forms.

### The Hwasun Seoyuri tracksite

**Figure 1:**
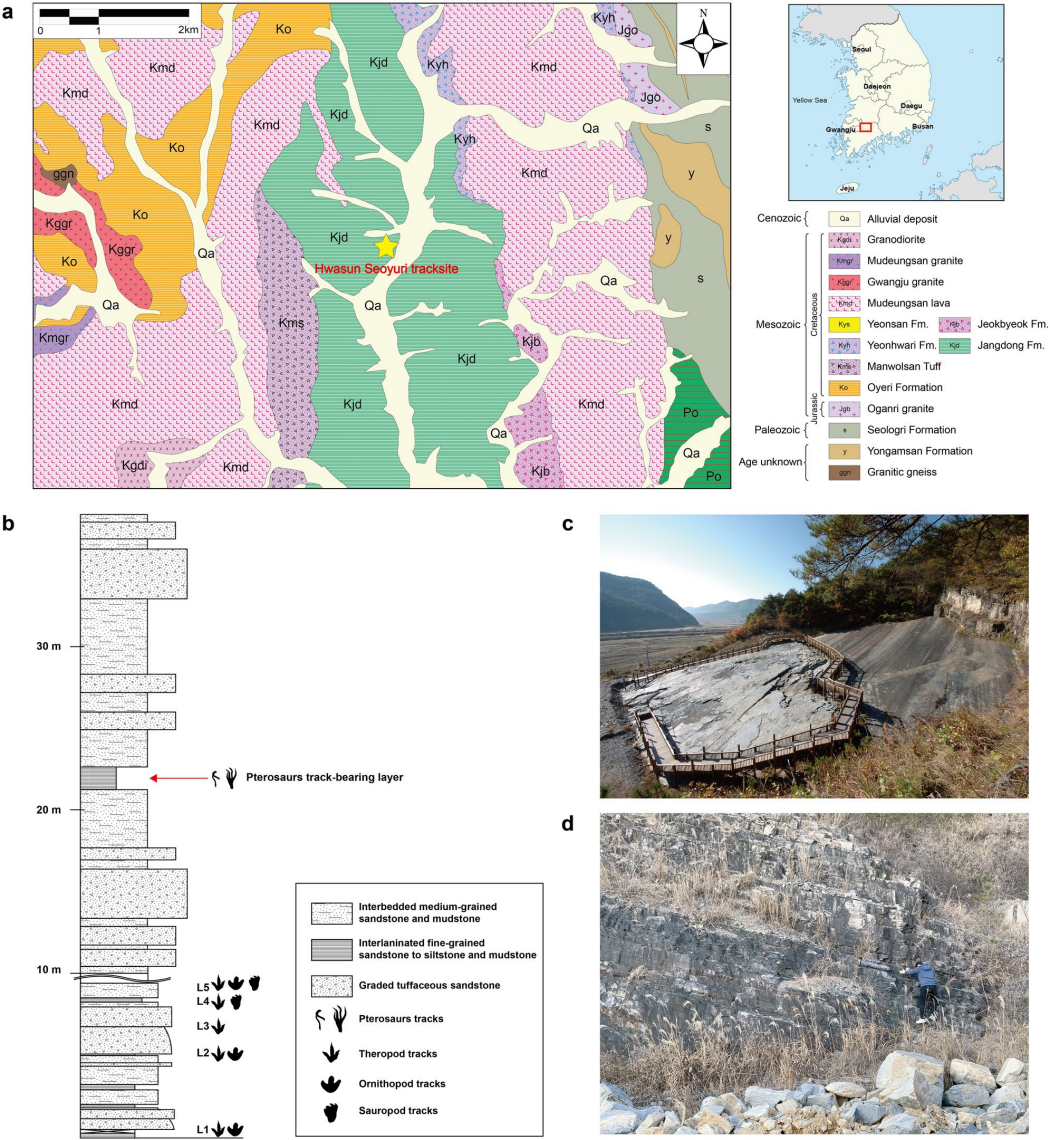
Location and columnar section of pterosaur footprint-bearing horizon. (**a**) Geological map of the Hwasun Seoyuri tracksite in the Jangdong Formation of the Neungju Basin in Korea (modified after Kim and Park^7^). (**b**) Columnar section of pterosaur and dinosaur track-bearing horizons. (**c**) Photograph of the Hwasun Seoyuri tracksite. (**d**) The outcrop of pterosaur footprint-bearing horizon. (**a**) and (**b**) were generated in Adobe Illustrator 2021 (https://www.adobe.com/illustrator).

The Neungju Basin is a non-marine sequence formed under the extensional or trans-tensional tectonic regime in the southwestern part of the Korean Peninsula^6^ (Fig. 1a). The basin comprises six formations composed of alluvial to lacustrine sediments with interbedded pyroclastic materials. In ascending order, these are (1) Oryeri Formation, (2) Manwolsan Tuff, (3) Jangdong Formation, (4) Jeokbyeok Formation, (5) Mudeungsan Lava, and (6) Ongam Conglomerate^7^. The Hwasun Seoyuri tracksite horizon lies within the Jangdong Formation, which consists predominately of silty mudstones and fine sandstones representing an alluvial plain and sandflat with pyroclastic materials^8^. Together with the footprints, various sedimentary structures, including desiccation cracks, ripple marks, and sulfate and halite casts, suggest that this formation was deposited under arid to semi-arid conditions^9^. The age of the Jangdong Formation is inferred to be 94 Ma based on the U–Pb dating of zircons^8^. According to the International Commission of Stratigraphy, this indicates the latest Cenomanian age of the Jangdong Formation^10^.

Pterosaur tracksites of comparable (Cenomanian) age to the Hwasun Seoyuri tracksite are restricted to the Candeleros Formation in Argentina^11^, which has also produced pterosaur bones^12^, and several localities in the Dakota Group of Colorado, USA^12,13^. The body fossil records for Cenomanian age pterosaurs are represented by an assemblage of well-preserved but fragmentary bones from the Lower Chalk of England (see Smith et al.^14^ and references therein) containing an ornithocheirid and at least one lonchodectids; a large and rapidly accumulating assemblage of often incomplete but uncrushed pterosaur bones from the Kem Kem Group of Morocco^15–23^ with several species of ornithocheirids and azhdarchoids based on jaw remains (Smith et al. in review); incomplete but associated skeletons of an istiodactylid (*Mimodactylus*), azhdarchoid (*Microtuban*), and an ornithocheirid from the Sannine Formation of Lebanon^24,25^; isolated bone fragments of an ornithocheirid, a coloborhynchid, and a lonchodectid from the Cenomanian of European Russia (e.g., ^26,27^); and a few fragments, possibly of azhdarchids, from the Khodzhakul Formation of Uzbekistan^28–31^.

The Hwasun Seoyuri tracksite has yielded about 1,500 dinosaur tracks that form more than 60 trackways distributed across five different horizons^32,33^. Sauropod and ornithopod trackways have been reported^34^, but most of these footprints are theropod tracks^32,33^ (Fig. 1b and c). These tracks also include an enigmatic theropod trackway^35^ and have been used to analyze the acceleration phase of a theropod^36^. The Hwasun Seoyuri tracksite was initially exposed during quarrying but is now the Republic of Korea’s Natural Monument No. 487 and forms part of the Mudeungsan UNESCO Global Geopark.

### Pterosaur footprints at the Hwasun Seoyuri tracksite

**Figure 2:**
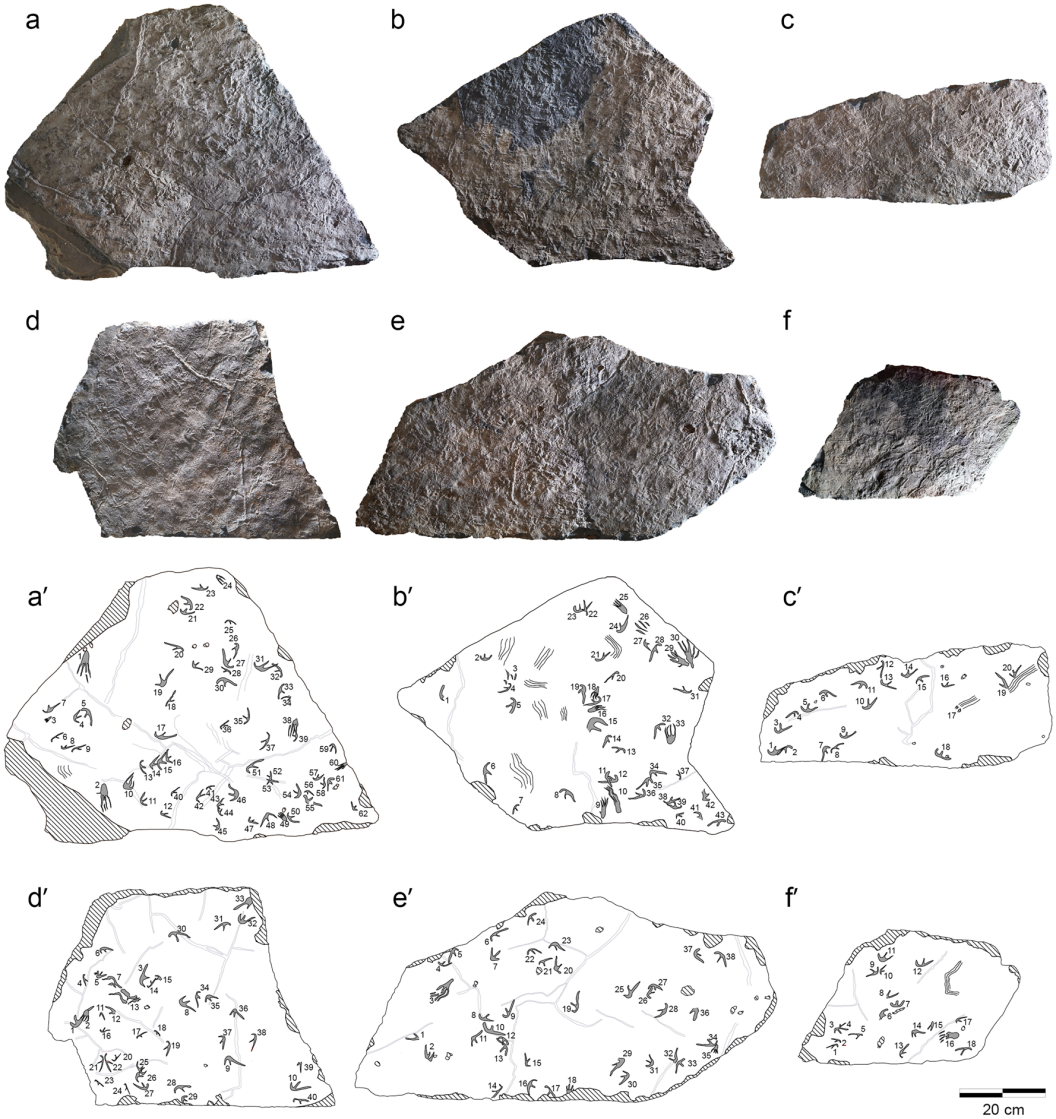
Photographs and interpretive drawings of pterosaur track-bearing slabs. **(a–f)** Photograph of KDRC-HW-PT01–06. **(a′–f′)** Interpretive drawing of KDRC-HW-PT01–06.

**Figure 3:**
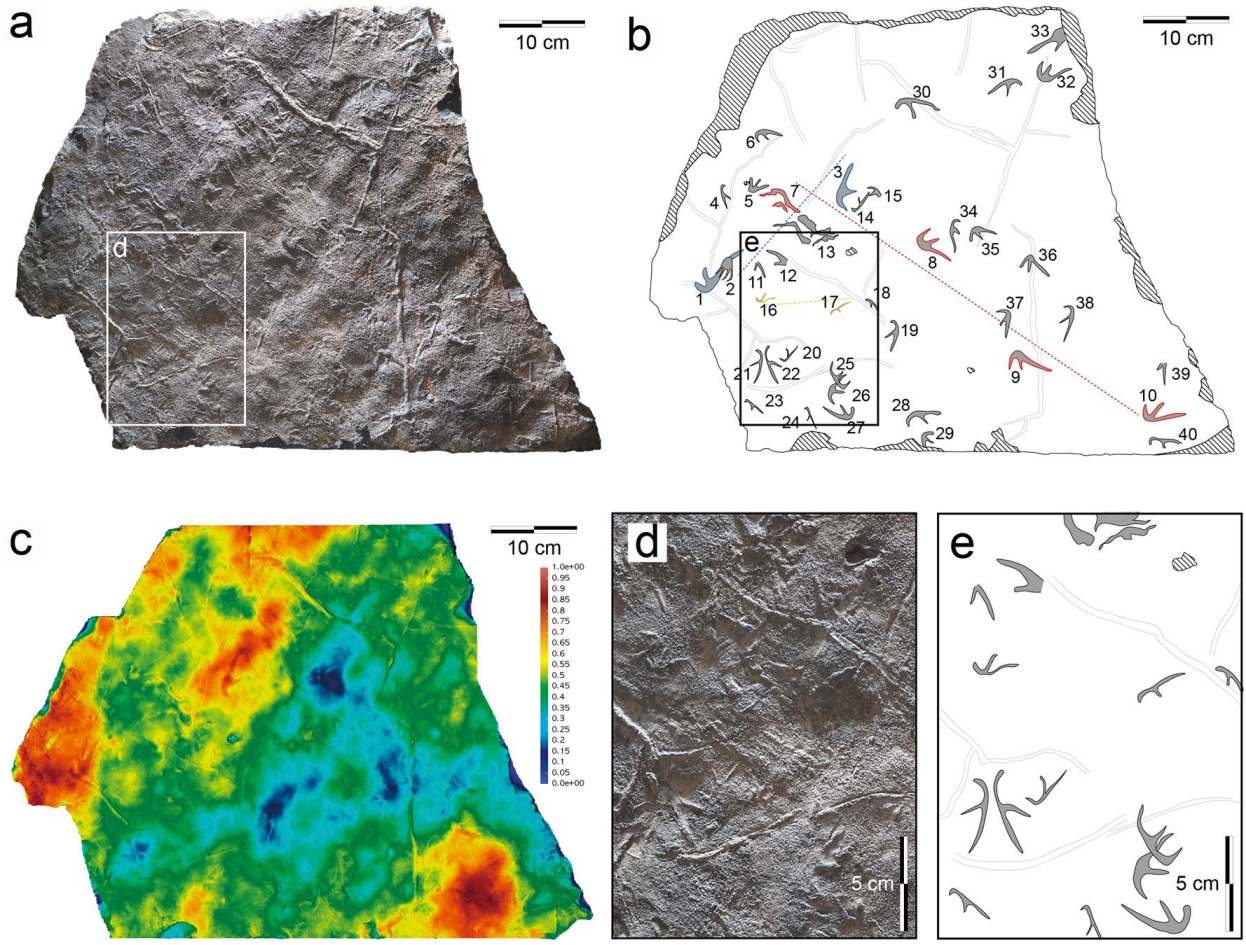
Photographs, 3D images, and interpretive drawings of KDRC-HW-PT04. **(a)** Photographs of KDRC-HW-PT04. **(b)** Interpretive drawing of KDRC-HW-PT04. **(c)** Color depth map of KDRC-HW-PT04. **(d)** Close-up photograph of KDRC-HW-PT04. **(e)** Close-up interpretive drawing of KDRC-HW-PT04.

Hundreds of pterosaur footprints, mainly of manus, were found on ten isolated slabs (KDRC-HW-PT01–PT10) originating from a single bed (Fig. 1d) at the outcrop of the Hwasun Seoyuri tracksite (Fig. 2). The total number of pterosaur footprints is more than 300, but in this study, we focus on a more restricted set of 221 footprints (198 manus and 23 pes) located on slabs KDRC-HW-PT01–PT06 (Suppl. Table S1). The total area of the six slabs is 1.56 m^2^. Footprint density ranges from 109 per m^2^ on slab KDRC-HW-PT05 (Fig. 2e and e′) to 175 footprints per m^2^ on slab KDRC-HW-PT01 (Fig. 2a and a′) with an average of 143 per m^2^ for all six slabs. Dense occurrences of footprints with some overprinted are present on slabs 1, 2, and 4 (Fig. 2). Apart from a sequence of four manus prints on KDRC-HW-PT04 (Fig. 3), we could not identify trackways on the other slabs, and most footprints appear to be randomly oriented. The anatomical details, such as skin or digital pad impressions, are not discerned because of subsequent evaporite traces.

Based on the criteria of Marchetti et al.^37^ describing the preservation level of vertebrate ichnofossil, the footprints in this study are hard to consider as [optimal; exceptional; elite] class because several tracks are overprinted, and diminutive secondary evaporite casts had erased the details features such as skin or digital pad. However, we considered it as [well; good; fine; sub-optimal] class in that they have “*fairly shape and clear footprints*” and “*manus prints distinguishable from pes prints*.”

**Figure 4:**
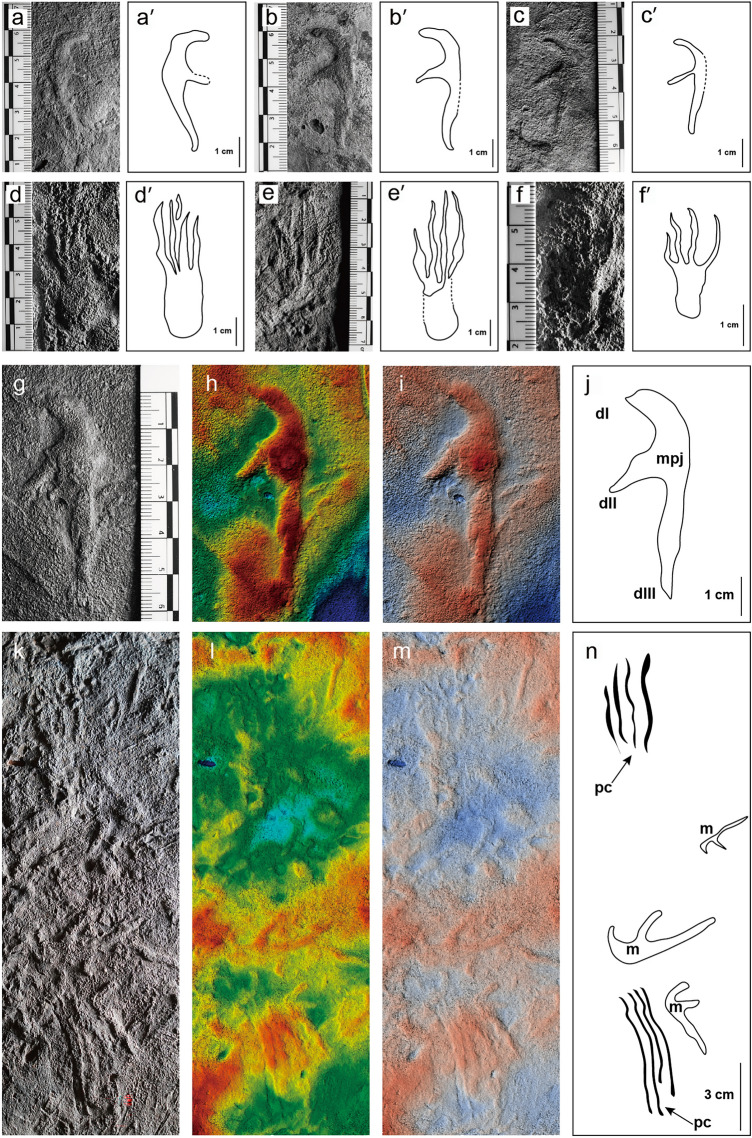
*Pteraichnus* isp. in the Hwasun Seoyuri tracksite. **(a–c)** Photographs of *Pteraichnus* isp. manus imprints. **(a′–c′)** Interpretive drawing of *Pteraichnus* isp. manus imprints. **(d–f)** Photographs of *Pteraichnus* isp. pes imprints. (**d′–f′)** Interpretive drawing of *Pteraichnus* isp. pes imprints. **(g–j)** Photograph, 3D images, and interpretive drawing of *Pteraichnus* isp. manus imprint on KDRC-HW-PT04. **(k–n)** Photograph, 3D images, and interpretive drawing of pes claw marks on KDRC-HW-PT03. *dI-III*, digits I*-*III; *mpj*, metacarphophalangeal joint; *pc*, pes claw marks; *m*, manus impression.

The manus imprints are typically pterosaurian: tridactyl, digitigrade, and asymmetric (Fig. 4a–c and 4a′–c′) with three digits radiating from a metacarpophalangeal joint. Small footprints (manus length < 30 mm) tend to be of even depth, but in medium to large footprints (Fig. 4g-j), the impression of the metacarpo-phalangeal joint is deeper than the impression of the digits. Skin impression is not observed in the manus imprints, but, as noted, this may reflect preservational bias rather than true absence.

**Figure 5:**
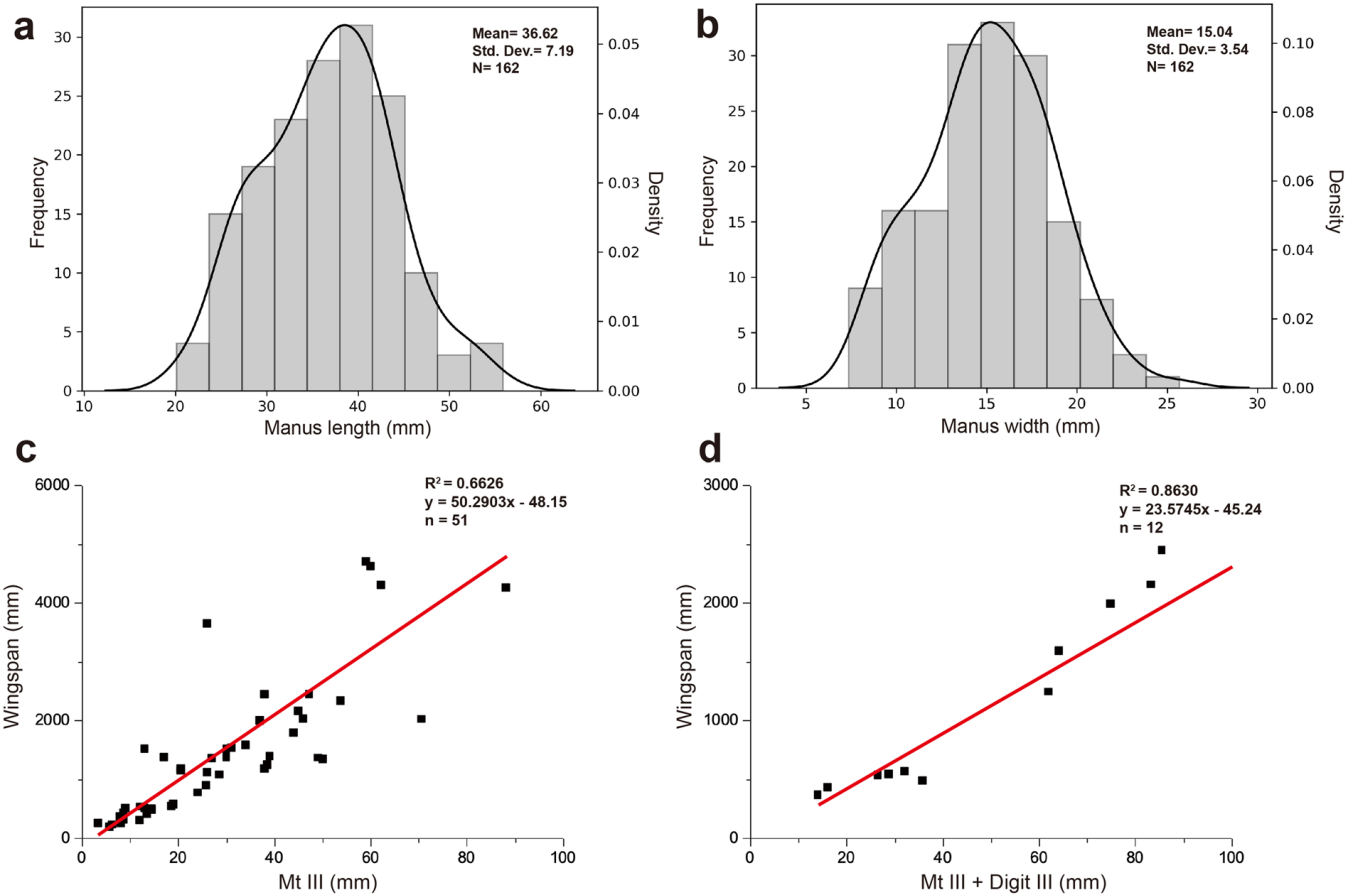
Frequency histogram with density curve of the manus length (**a**) and width (**b**) data. Scatter plots and linear regression of Metatarsal III **(c)** and Metatarsal III + Pes digit III **(d)** versus wingspan. This analysis was performed based on Jamovi (v.1.6.23.0) and drawn through Python (v.3.8.8) and Adobe Illustrator (2021).

Manus prints vary in length from 21.50 to 61.15 mm with a mean value of 36.67 mm (Fig. 5a) and vary in width from 6.83 to 25.67 mm with a mean value of 15.04 mm (Fig. 5b). The length/width ratio varies from 1.60 to 3.79 with a mean value of 2.57. The lengths of digit I and digit II show a close correspondence, also reflected in mean values (Digit I: x̄ = 14.06 mm, Digit II: x̄ = 14.12 mm). By contrast, digit III is always longer than the digits I and II (Digit III: x̄ = 26.07 mm). In terms of their digit width, small footprints (length less than 30 mm) are narrow and slender (Fig. 3d and e) though this may reflect the collapse of the substrate^38^. The tip of Digit I is directed anterior-laterally with a gentle inward curve, while digit II is directed posterior-laterally. Digit III is oriented posteriorly, and unlike some pteraichnites, there is no digit IV impression in any of the footprints. Divarication between digit I and digit II ranges from 27.35° to 122.78° with a mean value of 74.57°. Between digit II and digit III, it ranges from 20.77° to 90.63 with a mean value of 44.83°.

The pes imprints are much rarer (about 10% of the total) than the manus imprints (Fig. 4d–f and d′–f′). They exhibit a typically pterosaurian morphology: asymmetric, plantigrade, and tetradactyl. Pes imprints are elongated and sub-rectangular in shape and lack any evidence of skin texture. Pes length ranges from 27.38 to 64.35 mm with a mean value of 47.93 mm. Pes width ranges from 13.73 to 32.37 mm with a mean value of 20.40 mm. The length/width ratio of the pes varies from 1.59 to 2.95 with a mean value of 2.19. Pes imprints are, on average, longer than manus (mean value = 1.31). Digits II, III, and IV are of sub-equal length (Fig. 4f) or show a small increase in length from II to III (Fig. 4d and e), while digit I is always markedly shorter. Digits I–IV are usually sub-parallel and exhibit low divarication angles. Impression of pedal phalanges and claw marks composed of up to four parallel lines are found on some slabs (e.g., Fig. 4k–n).

The footprints in this study, especially the pes imprints, might seem to share some morphological features of crocodilian tracks at a glance (e.g., *Antipus*, *Batrachopus*, and *Crocodylopodus*). However, there are cogent reasons to consider them pterosaurian footprints, not crocodilian. Firstly, the manus in the Hwasun Seoyuri is tridactyl, and it is very different from a crocodilian manus with pentadactyl. Although the latter could be preserved only three digits depending on their locomotion or preservational condition, many manus footprints that show clear three digits boundaries refute this possibility. Secondly, the details of pes morphology of Hwasun Seoyuri are different from those of previously reported Mesozoic crocodilian. Among the ichnotaxa known as the crocodilian walking track in the Mesozoic, *Antipus*, *Batrachopus*, and *Crocodylopodus* can be considered to share some form of the pes^39^. However, slender digits and low divarication among the digits (almost parallel) distinguish it from the crocodilian ichnotaxa, as mentioned above, although they are features often found in pterosaurian footprints. Overall, it is more considerable to see the footprints in the Hwasun Seoyuri tracksite were attributed to pterosaurian, not crocodilian.

### Identity of the Hwasun Seoyuri pteraichnites

To date, five pteraichnite ichnogenera have been described: (1) *Agadirichnus*^40^; (2) *Pteraichnus*^41^; (3) *Purbeckopus*^42^; (4) *Haenamichnus*^43^; and (5) *Rhamphichnus*^44^. Several diagnostic characteristics of *Agadirichnus* make it distinguishable from the Hwasun Seoyuri pteraichnites, such as its much larger size, a relatively short, rounded manus digit I, a relatively elongated metatarsus (Digit/Metatarsus ratio = 0.4) and the presence of claw marks but absence of digit traces in some pes imprints^45^. Pteraichnites in this study are smaller, manus digits I and II are subequal in length, and the average D/Me ratio = 1.12. *Haenamichnus* prints with an average pes length of > 200 mm are at least four times the principal dimensions of the Hwasun Seoyuri footprints and distinguished from the latter by the highly elongated, narrow profile of the pes prints^43^. The *Purbeckopus*^42,46^ are also more significant than those at the Hwasun Seoyuri tracksite and further distinguished by the subtriangular outline of the pes print, which also has a slight medial curvature, and the relatively wide impressions of the digits. The latter contrast sharply with the much narrower and relatively straight pedal digits of the Hwasun pes imprints. *Ramphichnus* exhibits a range of characters that clearly distinguish this print morphotype from the Hwasun Seoyuri footprints. These include anteriorly directed manus prints, a pentadactyl pes print, and a D/Me ratio that often exceeds 2.0^44^.

Following its initial definition by Stokes^41^, the diagnosis of *Pteraichnus* has been developed and amended by Lockley et al.^47^ and Billon-Bruyant and Mazin^48^ to read as follows: quadrupedal tracks with digitigrade, tridactyl, elongated, and asymmetric manus imprint; anterior to anterolateral digit I, anterolateral to posterolateral digit II and posterior digit III; plantigrade, tetradactyl, elongated and subtriangular pes imprint; the length of digit II and III is slightly longer than digits I and IV. The prints at the Hwasun Seoyuri tracksite exhibit all these features and, amongst all known pteraichnites, both manus and pes prints correspond most closely, in terms of their morphology, to ‘classic’ examples of *Pteraichnus* currently assigned to *P*. *saltwashensis* and *P*. *stokesi*. Consequently, the Hwasun Seoyuri pteraichnites are formally assigned here to *Pteraichnus*.

Eleven ichnospecies of *Pteraichnus* have been named to date: *Pteraichnus saltwashensis*^41^; *P*. *stokesi*^47^; *P*. *palacieisaenzi*^49^; *P*. *parvus*^50^; *P*. *longipodus*^51^; *P*. *yanguoziaensis*^52^; *P*. *koreanensis*^53^; *P*. *nipponensis*^54^; *P*. *dongyangensis*^55^; *P*, *wuerhoensis*^56^; and *P*. *gracilis*^57^. Several of these, *P*. *saltwashensis*, *P*. *parvus*, *P*. *nipponensis*, *P*. *longipodus*, *P*. *dongyangensis*, *P*. *koreanensis,* and *P*. *gracilis* are clearly distinguished from the Hwasun Seoyuri footprints in that they have a relatively long metatarsus and relatively short digits with a Digit/Metatarsus ratio that is considerably less than 1.0 (0.38–0.73)^56^. In addition, the Hwasun Seoyuri footprints are also distinguished from these seven morphotypes by the following features: the similarity in the length of manus digits I and II; the curvature of manus digit I; and the relative shortness of pes digit I. These features also distinguish the Hwasun Seoyuri footprints from prints of *P*. *stokesi*, *P*. *wuerhoensis*, *P*. *palacieisaenzi*, and *P*. *yanguoziaensis*. The latter two forms are much larger (manus and pes length > 10 cm) than the Hwasun Seoyuri prints.

At present, the Hwasun Seoyuri footprints cannot be assigned to any of the currently known forms of *Pteraichnus*. Taxonomy of pteraichnites, especially *Pteraichnus*, is currently in flux with debate over the validity of some forms^48,58^ and difficulties with the composition of some ichnogenera. For example, as we have shown above, some morphotypes of *Pteraichnus* are markedly different from each other, most strikingly in terms of the D/Me ratio. Until greater clarity and maturity of pteraichnite taxonomy is achieved, we prefer not to erect another new form of *Pteraichnus* and so, for the present, refer to *Pteraichnus* isp.

### The identity of the trackmaker

To determine the size of the trackmakers that generated the Hwasun Seoyuri footprints, we compiled a morphometric data set consisting of measurements for the length of the metatarsal III, the combined lengths of metatarsal III + pedal digit III, and the wingspan (equivalent to 2.1 × forelimb length). This dataset (Suppl. Table S2) comprises 51 specimens representing 33 species and 12 of the 16 principal clades of pterodactyloid pterosaur. Equations relating wingspan to the length of the metatarsal III and the combined length of the metatarsal III + pedal digit III (equivalent to the ‘pes’ in pteraichnites) were generated from linear regressions of bivariate plots for these values conducted using Excel (v.16.58; 2022) (Fig. 5c and d). Substituting values for pes length and metatarsal III length derived from the Hwasun Seoyuri footprint data allowed us to generate estimates of the wingspans for the smallest (0.54–0.56 m), largest (1.25–1.48 m), and average-sized trackmakers (1.07 m).

The last two decades have seen a rapid increase in our knowledge of pterosaur skeletal anatomy, thanks primarily to a series of discoveries in the Upper Jurassic of China (see Zhou et al.^59^ and references therein) and the Lower Cretaceous of China (e.g.^60^) and Brazil (see Beccari et al.^61^ and references therein). Consequently, the manus and pes details are now known as representatives of all four major pterodactyloid groups: ornithocheiroids; ctenochasmatoids; dsungaripteroids; and azhdarchoids, and thirteen of the sixteen principal clades.

The manus-only trackway in the KDRC-HW-PT04 (red dotted line in Fig. 3b) shows that the manus was fully laterally rotated during terrestrial locomotion. Because these are diagnostic features of pterodactyloid rather than non-pterodactyloid pteraichnites, consequently we restrict comparisons to pterodactyloid pterosaurs^44^. There are four characteristics of the Hwasun Seoyuri footprints that permit direct comparison with the manus and pes of body fossils: (1) relative lengths of the manus digits; (2) relative lengths of the pes digits; (3) relative size of the manus and pes prints (specifically, length of manus digit III compared to the total length of the pes print); (4) the Digit III/Metatarsus (D/Me) ratio. Among these characteristics, the relative size of the manus and pes prints needs to be considered more carefully than other factors. The pterosaur trackway consisting of both manus and pes is not found in this study. Therefore, we used the average proportion of manus and pes sizes of all samples instead. However, since the preservational condition may vary depending on the size of individuals, this ratio might be tentative. Thus, it can be supplemented if a trackway with completely preserved manus and pes prints is found in further study. To determine the general identity of the Hwasun Seoyuri printmaker, we compared these four characteristics with the same characters of well-preserved examples of body fossils for representatives of each of the thirteen principal clades of pterodactyloids where the anatomy of the manus and pes is known.

If we can suppose the average size of the manus and pes can represent the actual size, Ornithocheiroids (including istiodactylids, ornithocheirids, pteranodontids, and nyctosaurids) can be excluded from consideration as trackmakers because the manus length (represented by manus digit III) is comparable to, or exceeds, pes length in ornithocheirids (e.g., *Zhenyuanopterus*^62^) and pteranodontids (*Pteranodon*^63^). This unusual proportion seems to apply to istiodactylids, based on *Haopterus*^64^ and *Mimodactylus*^24^, where the metatarsus is preserved but not the pedal digits. Nyctosaurids lack manus digits I-III (e.g.^65^), which excludes them from further consideration.

In ctenochasmatoids, the Digit/Metatarsus ratio is less than one in all species for which data is available. Moreover, the two principal clades that are known to have persisted to at least the end of the Lower Cretaceous (lonchodectids and ctenochasmatids) and thus might be considered as potential trackmakers for the Hwasun Seoyuri prints exhibit some of the lowest D/Me ratios for any pterodactyloid (*Gladocephaloideus* = 0.66; *Pterodaustro* = 0.58).

**Figure 6:**
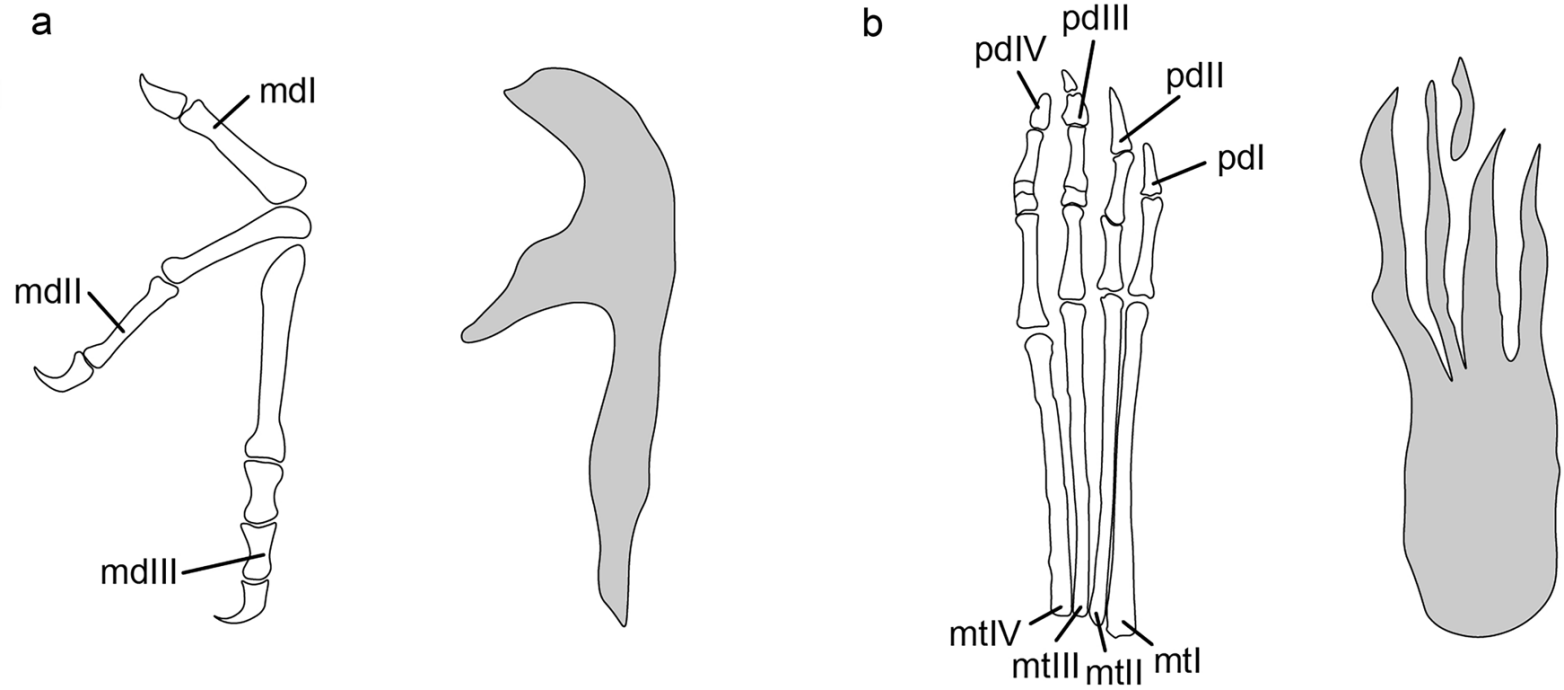
Comparison of pterosaur manus and pes skeletons with *Pteraichnus* isp. in the Hwasun Seoyuri tracksite. **(a)** Manus skeleton outline drawing of *Noripterus complicidens* (redrawn from Hone et al.) and manus imprint of *Pteraichnus* isp. in the Hwasun Seoyuri tracksite. **(b)** Pes skeleton outline drawing of *N. complicidens* (redrawn from Li et al.) and pes imprints of *Pteraichnus* in the Hwasun Seoyuri tracksite. *mdI-III*, manual digits I-III; *mtI-IV*, metatarsalsI-IV; *pdI-IV*, pedal digitsI-IV.

The manus and pes of the basal dsungaripteroid *Germanodactylus* (SMNK PAL 6592) correspond closely to the Hwasun Seoyuri prints. Manus length is approximately 66% that of the pes, manus digits I and II are of similar length, pes digits II–IV are comparable in length, while pes digit I is somewhat shorter, and the D/Me ratio is approximately 1.0. The metatarsus of the pes prints seems somewhat broader and more robust, while the pedal digit impressions appear relatively narrow and elongated. However, these are minor differences and might be attributable to the presence of soft tissues in the former case and the nature of print formation in the latter. In a similar case, tiny manus imprints may be observed thinner than the actual due to the collapse of the substrate, and this phenomenon may occur in the digit of the pes imprint^38^. The main argument against a *Germanodactylus*-like trackmaker is that this taxon is Upper Jurassic in age, while the pes of Lower Cretaceous dsungaripteroids such as *Noripterus*, much closer in time and space to the Hwasun Seoyuri footprint assemblage, is distinctly different (see above) with, for example, a D/Me of 0.77^56^ (Fig. 6).

Azhdarchoids have relatively specialized pes, with relatively long metatarsus and short digits. The D/Me ratio = 0.66 in azhdarchids such as *Zhejiangopterus* (Unwin pers. obs.); 0.89 in chaoyangopterids such as *Jidapterus*^66^ and tapejarids ranges from 0.81 in *Tupandactylus*^61^ to 0.96 in *Sinopterus*^67^. This configuration is quite unlike the elongate digits and relatively short metatarsus of the Hwasun Seoyuri trackmaker. Moreover, tapejarids such as *Sinopterus*^68^ and *Tupandactylus*^61^ pes digits I-IV are subequal in length.

At present, it would seem most likely that the Hwasun Seoyuri trackmaker was a dsungaripteroid. It is supported by the presence of dsungaripteroids in Asia in the Lower Cretaceous, the identification of a dsungaripteroid, *Noripterus*, as the trackmaker at the Huangyangquan Reservoir site^56^ and evidence that dsungaripterids such as *Noripterus* and *Dsungaripterus* were terrestrial feeders^69^. However, the highest congruence between prints and skeletal morphology is with *Germanodactylus,* an Upper Jurassic dsungaripterid exclusively known from Europe. Dsungaripteroids are not known to have persisted in Asia beyond the mid-Lower Cretaceous^70^, and the Hwasun prints are distinctly different (see above) from those of *Pteraichnus wuerhoensis*. Therefore, we conclude that the Hwasun prints were made by a relatively small dsungaripteroid as yet unknown from the body fossil record.

## Discussion

### Manus dominated footprint assemblage

The pteraichnites at the Hwasun Seoyuri tracksite are dominated by manus imprints. While this is unusual, manus-dominated print assemblages have been reported in the Summerville Formation of Utah^47^, the Hekou Group of China^71^, or Anza of Morocco^72^. There are two principal explanations for this unexpected pattern. The first is differential loading of the manus and pes during print formation^73–75^. In pterodactyloids, the effective center of mass of the body is located between or just posterior to the shoulder girdles^76^. Consequently, a more significant portion of the body mass was transmitted through the forelimbs during terrestrial locomotion than the hindlimbs^73–75^. Thus, loads imposed on the sediment surface by the manus were much higher than those imposed by the pes. Moreover, the ventral surface area of the manus appears to have been significantly less than that of the pes. These combined factors meant that the manus was more likely to leave an impression than the pes, especially on highly cohesive surfaces^75^. Where sediment surfaces admitted both manus and pes left impressions, the manus was likely significantly more profound than the pes. Not only this, there was a much greater likelihood of the manus leaving an undertrack rather than the pes^75^. However, in the case of the footprints at the Hwasun Seoyuri tracksite, it seems unlikely that the preponderance of manus prints reflects undertracks as the prints are not diffused, suggesting that they were made on the surface of the supersaturated or subaquatic substrate.

While asymmetry in loading might explain the predominance of manus imprints at the Hwasun Seoyuri tracksite, two assemblage features are inconsistent with this idea. First, manus impressions are more preserved irrespective of their size. Assuming the substrate was uniform in condition, the size of the foot was proportional to the overall size of the pterosaur, and the volume of the body was proportional to the cube of the pterosaur foot size, the load exerted by the largest manus (about 2.77 times that of the smallest) was approximately 21 times that exerted by the smallest manus. Even assuming a high degree of asymmetry between the loads exerted by the manus and pes, it seems surprising that the pedes of the largest individuals did not leave far greater numbers of impressions since they are likely to have exerted a load equivalent to or greater than that exerted by the manus of the smallest individuals. In direct contrast to this prediction, the 23 pes prints span a size range comparable to the manus and show a fairly even distribution. Moreover, slab KDRC-HW-PT04 bears only a single pes impression alongside 40 manus imprints, including the smallest examples (Fig. 3d and e).

Secondly, if the hypothesis of asymmetry in loading is correct, then the rare occurrences of pes prints might reflect localized decreases in sediment stiffness that allowed the pes to register on the substrate. However, we would expect pes impressions to occur in clusters, yet they show a nearly random distribution across the six slabs (Fig. 2). At present, asymmetry in loading provides only a partial explanation for the relative rarity of pes prints in the Hwasun Seoyuri print assemblage. Furthermore, this explanation does not address other prominent features of the print assemblage, such as the complete domination of the assemblage by random prints that do not appear to form trackways.

The possibility that the trackmakers were wading in the shallow water and that most prints were formed in a subaqueous environment provides an alternative explanation for the preponderance of manus prints and all prints’ random distribution and orientation. Lockley and Wright^77^ suggested that manus-dominated prints and partially preserved or scratch-like ungual marks might be attributed to behavior whereby pterosaurs contacted the surface of a subaquatic substrate with the manus while paddling with the hind limbs. Several of the slabs described in this study bear scratch marks that appear to have been made by unguals (Fig. 4k–n). Moreover, ripple marks and the absence of desiccation marks are consistent with the idea that the print-bearing surface was a shallow lake margin that was primarily subaquatic and only briefly exposed. Moreover, the greater length of the forelimb in pterodactyloid pterosaurs and, as discussed above, proportionally large body mass supported by the forelimbs during ‘terrestrial’ locomotion are likely to have led to a preponderance of manus, over pes, tracks. In addition, periods of total buoyancy would have allowed pterosaurs to generate random prints via occasional contact with the substrate by the manus (or very rarely by the pes) and intended to help in steering or stabilization.

The pterosaur track record provides numerous examples of pterosaurs generating footprints on sediments bordering aquatic environments: marine, lacustrine, and riparian^78^. Also, the interpretation of some clades, such as the ctenochasmatids as filter-feeding waders^69^, provides numerous potential opportunities for facultative aquatic locomotion. Sites such as that at Hwasun Seoyuri appear to document this behavior but require further study to precisely determine the nature of aquatic locomotion in pterosaurs.

### Mixed-aged pteraichnite assemblage

**Figure 7:**
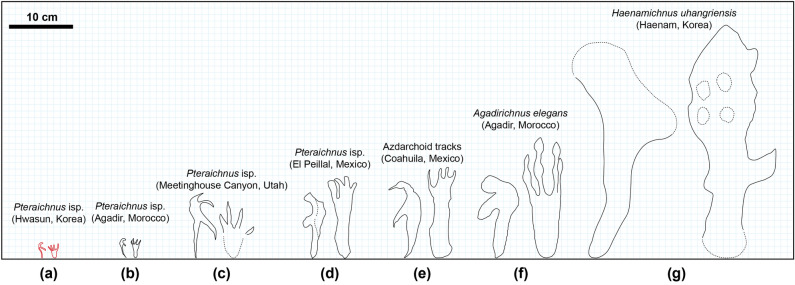
Comparison of *Pteraichnus* isp. in the Hwasun Seoyuri tracksite with other reported pterosaur footprints in the Late Cretaceous. **(a)** *Pteraichnus* isp. in the Hwasun Seoyuri tracksite (in this study). **(b)** *P*. isp. in the Tagragra Tracksite, Agadir, Morocco^45^. **(c)** *P*. isp. in the Blackhawk Formation, Meetinghouse Canyon, Utah^83^. **(d)** *P*. isp. in the Cerro del Pueblo Formation, El Peillal, Mexico^38^. **(e)** Azdarchoid tracks in the Las Encinas Formation, Coahuila, Mexico^81^. **(f)** *Agadirichnus elegans* in the Tagragra Tracksite, Agadir, Morocco^45^. **(i)** *Haenamichnus uhangriensis* in the Uhgnari Formation, Haenam, Korea^43^.

Several pteraichnite localities have been reported from the Upper Cretaceous (Fig. 7). At most locations, the pteraichnites are of medium to large size (> 10 cm)^38,43,45,72,79–83^. However, small to medium-sized pterosaur tracks have been reported from the North Horn Formation of Utah^84^ and the Tagragra tracksite near Agadir, Morocco^45^. We can now add the Hwasun Seoyuri tracksite, where the footprint assemblage was generated by multiple individuals with wingspans ranging from about 0.5–1.5 m. As discussed in Supplementary Information, the size distribution of prints forming the Hwasun Seoyuri pteraichnite assemblage is congruent, in relative terms, with size distributions for body fossil assemblages composed of multiple individuals. In the latter case, individuals at or near the lower bound of the size range tend to exhibit numerous features of osteological immaturity. Individuals of mean or modal size show a mix of mature and immature features, while those at or near the upper size bound present few features of immaturity^85,86^. The exact ontogenetic status of these stages is uncertain, though it seems likely that they represent juveniles, sub-adults, and adults (Suppl. Info.). Assuming that the shape and relative dimensions of the size distributions for the prints and body fossils are indeed more or less congruent, it would seem that the Hwasun Seoyuri assemblage represents a mixed age group probably composed of juveniles, sub-adults, and adults.

Several size distribution plots for body fossils, including *Rhamphorhynchus*, *Pterodactylus* (Suppl. Info. Fig. S1), and *Pterodaustro,* show bimodal distribution. In each case, the smaller mode consists of a relatively small number of individuals, only one quarter, one-fifth, or less the size of the largest individuals, and osteologically highly immature. In the case of *Pterodaustro,* the smallest individuals are equivalent in size to an embryo reported for this taxon^87,88^. This second ‘smaller’ mode consisting of very early juveniles, ‘flaplings’^2^, seems absent at the Hwasun Seoyuri tracksite. While this might be interpreted as evidence for habitat partitioning in pterosaurs, this conclusion should be treated with great caution, partially based on negative evidence.

It is tempting to argue that the remarkably high density of prints in the print assemblage (average density = 143/m^2^; highest density on a single slab = 175/m^2^) reflects gregarious behaviour^89^ and is generated by a ‘flock of pterosaurs’ composed of individuals of different sizes and, as argued above, of different ages. This possibility cannot be excluded, but it is also possible, if the print bearing surface persisted for any length of time, that the assemblage represents the accumulation of visits by single or small numbers of individuals to this location with little or no social interaction. It is not possible to distinguish between these alternatives, either for the Hwasun Seoyuri site or for other localities^54,56,57^.

## Conclusion

Small pterosaur footprints are preserved from the Upper Cretaceous Jangdong Formation of the Neungju Basin, Korea, where abundant dinosaur trackways have been reported. These footprints are assigned to *Pteraichnus* isp. and are some of the smallest pterosaur footprints in the Late Cretaceous. Manus-dominated assemblage has been interpreted as an imbalance of body mass and load per unit area. Otherwise, the lack of pes might be caused in shallow water during the trackmakers were wading affected by buoyancy force given the randomly oriented footprints and claw marks. The high density of variable-sized footprints could be inferred as gregarious behavior of a pterosaur multi-aged flock(s). The morphology of the footprints is somewhat congruous with dsungaripteroid. However, the difference in detail shape and the absence of fossil record in the Late Cretaceous of Asia suggest that the pterosaur footprints in the Hwasun Seoyuri tracksite were attributed to small dsungaripteroid as yet unknown from Asia.

## Supplementary Information

Supplementary Information 1.

Supplementary Information 2.

Supplementary Information 3.
