# Supplementary material for: Evidence for a mixed-age group in a pterosaur footprint assemblage from the early Upper Cretaceous of Korea
Source: Sci Rep. 2022 Jun 23;12:10707. doi: 10.1038/s41598-022-14966-5 (PMC9226182; doi:10.1038/s41598-022-14966-5)
Supplement: Supplementary file 1 — Supplementary Information 1. [file 41598_2022_14966_MOESM1_ESM.docx]

**Supplementary Information**

**Evidence for a mixed-age group in a pterosaur footprint assemblage from the early Upper Cretaceous of Korea**

Jongyun Jung^1^, Min Huh^1,*^, David M. Unwin^2^, Robert S. H. Smyth^3^, Koo-Geun Hwang^4^, Hyun-Joo Kim^5^, Byung-Do Choi^6^, Lida Xing^7^

^1^ Department of Geological and Environmental Sciences & Korea Dinosaur Research Center & Mudeungsan Geotourism Center, Chonnam National University, Gwangju, 61186, Republic of Korea

^2^ School of Museum Studies, University of Leicester, 19 University Road, Leicester, LE1 7RF, UK

^3^ School of Geography, Geology and the Environment, University of Leicester, University Road, Leicester, LE1 7RH, UK.

^4^ Yeosu Samil Middle School, Yeosu, 59655, Republic of Korea

^5^ Division of Earth and Environmental System Sciences, Pukyong National University, Busan, 48513, Republic of Korea

^6^ Daegu National Science Museum, Daegu, 43023, Republic of Korea

^7^ School of the Earth Sciences and Resources, China University of Geosciences, Beijing 100083, China.

^*^ minhuh@jnu.ac.kr

**Materials and Methods**

The pterosaur footprints appear to be restricted to a single horizon above the dinosaur tracks bearing beds at the Hwasun Seoyuri tracksite (Fig. 1d). Most of the pterosaur footprints described in this study were found on a series of ten isolated slabs, but several different footprints were discovered at the outcrop. These isolated slabs originated from the single layer since they have the same thickness, sedimentary features on the top and bottom surfaces, and vertical sections. All pterosaur footprint-bearing slabs were deposited in the Korea Dinosaur Research Center, Chonnam National University, Gwangju, Korea, under the designated acronym KDRC-HW-PT.

Footprints on the slabs are preserved as a positive hyporelief at the junction of laminated, tuffaceous, fine sandstone-mudstone couplets. The slab surfaces are more or less flat or slightly undulating. Vertical sections of the slabs reveal fine sandstone to mudstone with flaser or wavy-bedding.

We adopted the formalized terms for describing pterosaur footprints as proposed by Pascual Arribas and Sanz Péres^1^ and Billon-Bruyat and Mazin^2^ and gathered metric data for 221 footprints (Suppl. Table S1). Photographs of the slabs and footprints were taken with a digital camera SONY alpha 7 Mark Ⅲ and digital lenses SEL80M29G and SEL2470Z. Initial drawings of the footprints were generated using Procreate (v.5.2.5) for iPad, and images were created in Adobe Photoshop (2021) and Adobe Illustrator (2021). The guidelines for creating 3D images were followed by Falkingham et al.^3^ and Romilio^4^. Photographs were converted into the 3D models using Agisoft Metashape Professional (v.1.7.1) and visualized using Paraview (v.5.9.0).

**Profiling the Hwasun Seoyuri trackmakers**

**How many individuals?** Pterosaur footprints preserved on the six slabs from the Hwasun Seoyuri tracksite span a size range in which the largest manus imprints are 2.8 times the size of the smallest imprints, and the largest pes imprint is 2.35 times the smallest pes imprint (Figs. 5a & b; Supplementary Table S1). Multiple individuals were involved in the generation of this footprint assemblage. To estimate the minimum number of individuals required to generate the assemblage, we analyzed variation in footprint size for *Pteraichnus* *nipponensis*. Two trackways, ‘A’ and ‘B,’ consist of 24 and 18 prints. In trackway ‘A’ pes length varies by ±10% of the mean, while in track ‘B’ pes length varies by ±15% of the mean^5^. Applying these values to the Hwasun Seoyuri footprint assemblage indicates that at least four different size trackmakers would be required to generate the assemblage based on trackway ‘A’ variation. In comparison, trackway ‘B’ variation would need at least three trackmakers.

The availability of metric data and its variability from continuous series of prints forming trackways are more limited for other *Pteraichnus* morphotypes. In *P*. *wuerhoensis*, variation in pes length is approximately ±10% of the mean^6^, and illustrations of multiple *Pteraichnus* isp. trackways from the Upper Jurassic Crayssac assemblage^7^ also exhibit slight print length variation. Therefore, we are confident that the footprint assemblage at the Hwasun Seoyuri tracksite was generated by at least 3 to 4 individuals. Given the high density of the prints, the total number of individuals was likely to be much higher.

**How many species of pterosaurs?** We have shown that multiple individuals generated the Hwasun Seoyuri footprint assemblage. Therefore, it is critical to determine whether these individuals represent one or more trackmaker species. There are two lines of evidence, morphology and the size distribution of footprints, suggesting that there was a single species of trackmaker. The footprints’ most significant variation relates to (a) size, which is interpreted here as evidence for individuals of different growth stages, and (b) the degree of divergence between the manus digits. The divergence between digits I and II ranges from 27.35°–122.78° and between II–III from 20.77°-90.63°. This variability may reflect several factors, most importantly, variation, such as gait and locomotory style, and thus cannot be considered evidence for multiple trackmaker species. Moreover, close ranges in manus digit divarication angles have been reported in, for example, *Pteraichnus wuerhoensis*, where 114 prints show a similar size range to those from the Hwasun Seoyuri tracksite, are interpreted as having been made by a single species of pterosaur^6^.

Features of the Hwasun Seoyuri footprints representing skeletal morphology, such as the relative length of the digits and the digit/metatarsus ratio, show relatively slight variation. In addition, distinctive features of the footprints, such as the relatively narrow elongate pedal digit impressions, and medial curvature of manus digit Ⅰ, are ubiquitous in well-preserved footprints. One exception is a morphological difference in the width of the digits between the smallest and largest print size classes, but this may be reflected in sediment collapse immediately after print formation^8^.

Concerning size variation, data for manus (162 specimens) shows that the ratio of the maximum length compared to the minimum length is 2.77 and that for the width is 3.48. Individual slabs generally exhibit comparable size variability: maximum/minimum length ratios for the manus are 2.61. 2.60. 2.39, 1.80, 1.70, and 1.47. To determine whether this variability originated from a generation, we tested the normality of samples for the length and width of the manus. Both tests resulted in a unimodal distribution without any data transformation (Length: Shapiro-Wilk’s W = .991, p = 0.382; Width: Shapiro-Wilk’s W = .990, p = 0.342) (Figs. 5a & b). Díaz-Martínez et al.^9^ stated that when the footprint data show a normal distribution with unimodal, it can be estimated that it was attributed to a single population^10^, and interpreted variation within this distribution as the difference between multi-aged individuals. Thus, these results are consistent with the hypothesis that the Hwasun Seoyuri footprint assemblage represents a single population. The observation supports this conclusion that in the case of the print assemblage described by Li et al.^6^ and interpreted by them as having been generated by a single species of trackmaker, *Pteraichnus wuerhoensis*, maximum/minimum length and width ratios for the manus (2.71 and 2.17) and pes (2.13 and 2.26) are comparable to those for the Hwasun Seoyuri tracksite.

**Size variation of trackmakers and its ontogenetic significance**. In the pterosaur body fossil record, size plots for species represented by multiple (>10) individuals tend to exhibit unimodal or slightly bimodal distributions. The most standard size class is represented by individuals that show a significant degree of osteological maturity (e.g., *Pterodactylus*; Fig. S1). The smallest individuals, representing lower bounds of these distributions, tend to lack evidence of osteological maturity, such as fusion of epiphyses or fusion of compound skeletal elements^11-14^. In some cases, such as *Pterodaustro*, it may include hatchlings^15-17^. In a few species, including *Pterodactylus*, relatively high numbers of immature individuals may form the second mode in bimodal plots (Fig. S1). Most size distribution plots have an upper bound composed of rather large individuals who invariably exhibit high degrees of osteological maturity^11,12^.

In those species such as *Pterodaustro*, where samples appear to span almost the entire growth series from hatchling to relatively large mature individuals^18,19^, the latter are generally at least 5–6 times the linear dimensions of the former. However, the earliest perinatal growth stages are not preserved in most species. Typically, in these cases, such as *Pterodactylus* (Fig. S1), almost all individuals fall within a size equivalent to ± 50% of the mean size. These distributions include osteologically immature, partially mature, and, occasionally, fully mature individuals (Unwin pers. obs.).

The relative dimensions of the size distribution plots for the Hwasun Seoyuri footprints (Figs. 5a & b) show close correspondence to unimodal size distribution plots based on body fossils. Critically, print size spans a range equivalent to approximately ± 50% of the mean print length or width. We propose, therefore, that the Hwasun Seoyuri prints were most likely produced by a mixed-age group that included at least one or two immature individuals with approximately 0.55 in wingspan, at least one or two mature individuals that reached up to 1.5 m in wingspan, and multiple near mature/mature individuals with wingspans ranging from about 0.7–1.3m.

**Supplementary References**

1 Pascual Arribas, C. & Sanz Pérez, E. Huellas de Pterosaurios en el grupo Oncala (Soria, España). *Pteraichnus palaciei-saenzi*, nov. icnosp. *Estudios Geol.* **56**, 73-100 (2000).

2 Billon-Bruyat, J.-P. & Mazin, J.-M. The systematic problem of tetrapod ichnotaxa: the case study of *Pteraichnus* Stokes, 1957 (Pterosauria, Pterodactyloidae). *Geol. Soc. Spec. Publ.* **217**, 315-324 (2003).

3 Falkingham, P. L. *et al.* A standard protocol for documenting modern and fossil ichnological data. *Palaeontology* **61**, 469-480 (2018).

4 Romilio, A. *An instructional guide to visualising dinosaur tracks* (2020).

5 Lee, Y.-N., Azuma, Y., Lee, H.-J., Shibata, M. & Lü, J. The first pterosaur trackways from Japan. *Cretac. Res.* **31**, 263-273 (2010).

6 Li, Y., Wang, X. & Jiang, S. A new pterosaur tracksite from the Lower Cretaceous of Wuerho, Junggar Basin, China: inferring the first putative pterosaur trackmaker. *PeerJ* **9**, e11361 (2021).

7 Mazin, J.-M., Billon-Bruyat, J.-P., Hantzpergue, P. & Lafaurie, G. Ichnological evidence for quadrupedal locomotion in pterodactyloid pterosaurs: trackways from the Late Jurassic of Crayssac (southwestern France). *Geol. Soc. Spec. Publ.* **217**, 283-296 (2003).

8 Rodríguez-de La Rosa, R. A. Pterosaur tracks from the latest Campanian Cerro del Pueblo Formation of southeastern Coahuila, Mexico. *Geol. Soc. Spec. Publ.* **271**, 275-282 (2003).

9 Diaz-Martinez, I. *et al.* Multi-aged social behaviour based on artiodactyl tracks in an early Miocene palustrine wetland (Ebro Basin, Spain). *Sci. Rep.* **10**, 1099 (2020).

10 Hammer, Ø. & Harper, D. A. T. *Paleontological data analysis* (John Wiley & Sons, 2008).

11 Bennett, S. C. The ontogeny of *Pteranodon* and other pterosaurs. *Paleobiology* **19**, 92-106 (1993).

12 Bennett, S. C. Year-classes of pterosaurs from the Solnhofen Limestone of Germany: taxonomic and systematic implications. *J. Vertebr. Paleontol.* **16**, 432-444 (1996).

13 Bennett, S. C. A statistical study of *Rhamphorhynchus* from the Solnhofen Limestone of Germany: Year-classes of a single large species. *J. Paleontol.* **69**, 569-580 (1995).

14 Dalla Vecchia, F. M. Comments on Triassic pterosaurs with a commentary on the "ontogenetic stages" of Kellner (2015) and the validity of *Bergamodactylus wildi*. *Rivista Italiana di Paleontologia e Stratigrafia* **124** (2018).

15 Chiappe, L. M., Codorniú, L., Grellet-Tinner, G. & Rivarola, D. Argentinian unhatched pterosaur fossil. *Nature* **432**, 571-572 (2004).

16 Codorniú, L. & Chiappe, L. M. Early juvenile pterosaurs (Pterodactyloidea: Pterodaustro guinazui) from the Lower Cretaceous of central Argentina. *Can. J. Earth Sci.* **41**, 9-18 (2004).

17 Codorniú, L., Chiappe, L. & Rivarola, D. Neonate morphology and development in pterosaurs: evidence from a Ctenochasmatid embryo from the Early Cretaceous of Argentina. *Geol. Soc. Spec. Publ.* **455**, 83-94 (2018).

18 Chinsamy, A., Codorniú, L. & Chiappe, L. Developmental growth patterns of the filter-feeder pterosaur, *Pterodaustro guinazui*. *Biology Letters* **4**, 282-285 (2008).

19 Chinsamy, A., Codorniu, L. & Chiappe, L. Palaeobiological implications of the bone histology of *Pterodaustro guinazui*. *The Anatomical Record: Advances in Integrative Anatomy and Evolutionary Biology* **292**, 1462-1477 (2009).


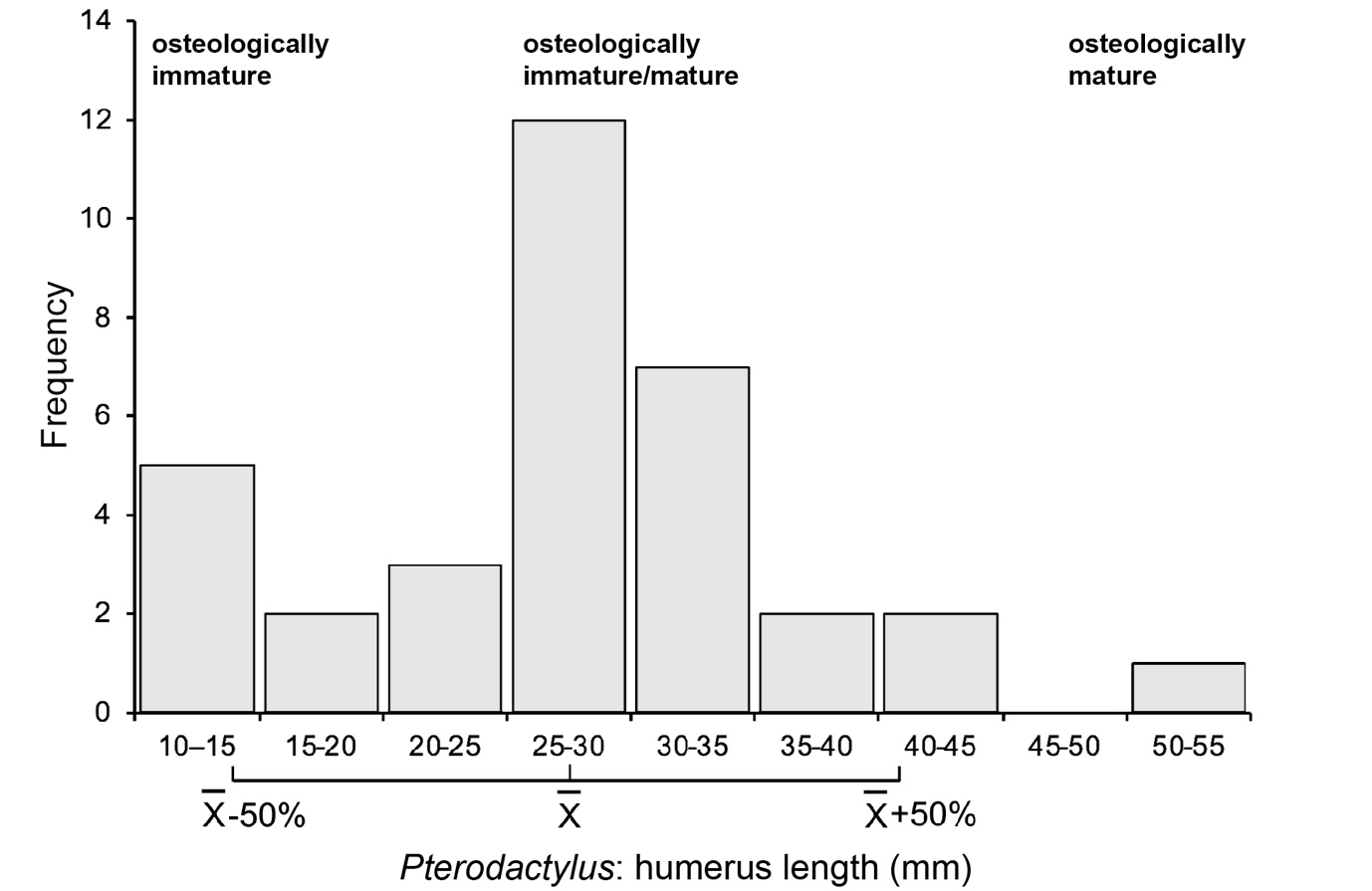


**Figure S1**. Frequency histogram of *Pterodactylus* humerus length. This analysis was performed based on Jamovi (v.1.6.23.0) and drawn through Python (v.3.8.8) and Adobe Illustrator (2021).
